# Two Cases of Parturition, with Remarks

**Published:** 1845-10

**Authors:** Jno. W. Richardson

**Affiliations:** Rutherford county


Art. IV.—*Two Cases of Parturition, with Remarks.* Read before the Medical Society of Tennessee, at its Annual Meeting, in May, 1845. By Jno. W. Richardson, M.D., of Rutherford county.

Case I.—On the night of the 28th of January last, I was summoned to see Mrs. Y. in her fourth labor. She had been the object of many remarks by her neighbors for more than two months, on account of her great size, the general conclusion from which was that she would have a plurality of children at her confinement. I had noticed her occasionally myself, and the enormous size of her abdomen had excited some uneasiness in my own mind, especially as I understood that I was to be her accoucheur.

When I arrived she was sitting by the fire, and we had rather an amusing conversation about her size, which was frequently interrupted, however, by the recurrence of labor pains. She said she had suffered immensely for several months, and looked to her confinement "with awful fears."

As the pains were pretty severe, and had commenced some six or eight hours before, I requested her to take the bed in order that I might make an examination. I first explored the abdomen, and have often wished since, that I had measured it; but as I did not, I will not venture to say how large I thought it was, lest I should be considered extravagant. A Dutchman once said of one of his fatted animals, that he was "*bigger lying down than standing up;*" and so it seemed with my patient.

On examining per vaginam the os uteri was found dilated to the extent of an inch or more, soft and yielding. The bag of waters presented finely, but I could touch no part of the child. I endeavored to quiet the apprehensions of my patient, but at the same time felt rather uncomfortable myself. After some two hours, the pains still recurring at regular intervals, and occasional vaginal examinations disclosing nothing more satisfactory than at first, with the exception of a slight advance of the waters, I determined to rupture the membranes. The consequence of this operation was a prodigious flow of liquor amnii. The woman was literally deluged, and although I commenced calling for sheets with which the most of the water was absorbed, still it ran through a thick quilt four double—through the bed, and some distance on the floor.

The abdomen was now soft, the skin wrinkled, and so pendulous, that it actually hung down over the pubis. I examined per vaginam again, but could feel no child. The uterus was quiet, and the woman expressed great relief. After some ten or fifteen minutes, the womb contracted freely, and I touched the child's head.

My anxieties were now all allyed, and the woman was shortly afterwards delivered of a *small* deformed child. She did well, and recovered rapidly.

*Remarks.*—Delivery was retarded, in this case, on account of the superabundance of liquor amnii, which had stretched the uterus so much as to destroy, to a considerable degree, its contractile power; and although the uterine contractions may have been as great as usual, still the great quantity of water must have destroyed much of their force, or in other words, have prevented their action on the child.

My experience is that a superabundance of liquor amnii is as bad for the child as a deficiency, and causes the woman much more pain in gestation.

Case II.—On the morning of the 31st of last January, at 4 o'clock, I saw Mrs. S., who was confined with her tenth labor. She had never had a physician with her in any previous confinement, but a considerable hemorrhage, which come on two or three hours before, had alarmed her so much, that she readily consented to a proposition from her husband to send for me.

Shortly after the commencement of the flooding she had labor pains, but they were feeble, and recurred at long intervals. An examination satisfied me that the bleeding was not sufficient to do any harm. The child's head presented, and the pains increased slowly. The os tincæ was open, very dilatable, and the waters were collecting finely. The hemorrhage recurred occasionally in small quantities until 9 o'clock, when she was delivered of a small male child, the presentation being the fifth of Baudelocque. The labor was easy, and there was no occasion for any interference. The smallness of the child excited my suspicion that the case might be one of twins, and accordingly, before I could tie the cord, and deliver the child to the nurse, the mother said, "Doctor there is another one." By placing my hand on her abdomen I discovered that she was correct. As soon as possible the cord was tied in two places, divided between the ligatures, and the child disposed of.

In a few moments the uterine pains returned, and I examined for the placenta, or another child. The placenta presented, and after unusual efforts was forced down so low in the vagina that I removed it. I then examined for the second child, but what should I find but another placenta presenting at the mouth of the womb? The uterus began to contract powerfully and almost without cessation. The placenta protruded considerably from the mouth of the womb, so as to lie in the cavity of the sacrum, but yet I could not remove it by such force as I felt justifiable in using.

Having learned nothing yet about the presenation of the child, I felt some uneasiness, though I expected, as is usually the case with twins, that the feet or breech would present, as the first had been a head presentation.

The loud complaints of the woman, her constant appeals for "help," and the incessant contraction of the uterus, rendered me more uneasy, and I determined to ascertain what was the source of the difficulty. I broke through the placenta with my finger, ruptured the membranes, and found a hand in the placental bag.

As it was impossible to remedy the position of the child, or even to ascertain its position correctly whilst the placenta remained, I determined to remove it, which I did pretty quickly, though not without leaving a large portion of the membranes behind.

An examination now disclosed the following state of things: the right hand near the vulva, both feet in the pelvic cavity, opposite the plane of the right ischium, and the head engaging the superior strait, after Baudelocque's first presentation.

As quick as possible, I caught hold of both feet with my left hand, and with the point of the index finger of the right hand against the head, I endeavored to chage the position of child, and deliver by the feet. Two efforts convinced me that it could not be done, for there had been no relaxation of the uterus from the instant the waters escaped, and every moment made the case more difficult. Grasping both feet as firmly as I could with the fingers of the left hand, I resolved on pushing them back into the uterus. Slowly, but firmly and resolutely, they were pushed up until they reached the brim of the pelvis, when by a little dexterity with the points of my fingers, they were sent back into the womb.

The head of the child advanced now rapidly, with the arm by its side, and the woman was delivered in two minutes of another male child. The second child was small, though larger than the first, and to my great mortification was born dead. It was alive at the birth of the first one, for the mother told me she felt it distinctly. The labor was over by 10 o'clock, and at 12 I left my patient doing as well as usual, expressing great obligations to me, and rejoicing that she had sent for "the Doctor."

*Remarks.*—This was a singular and interesting case, rendered so by the peculiar position of the second child. Was this the natural position of the child, or was it made to assume this position after the delivery of the first?

My opinion is that the second child changed its position entirely. I believe that it occupied the right side of the womb, with its back to the right lateral part of the abdomen of the mother, and that the first child lay in the left side of the uterus. When the first was delivered both placentæ were detached from the womb, and from some examination I was induced to think that they were slightly attached to each other. The placentæ came down between the second child and the left side of the uterus. The second child, I think, was made to change its position during the relaxation of the uterus, immediately after the birth of the first, the head following the course in which the placenta left the womb, and the breech thrown up nearly to the position which was occupied by the head. Had not this occurred the second child would have presented the breech, with the right buttock to the left acetabulum, the left to the right sacro-iliac symphisis, and the back to the right lateral region of the abdomen of the mother. These remarks are only conjectural, and you can take them for what, upon examination, they may appear to be worth.

It is certain that the child could not have been delivered if it had been permitted to remain in the position in which I first found it, with one arm, both legs, and the head all engaged in the superior strait.

Such a position would have sounded to me rather fanciful before I saw it, and it may perhaps seem so to you. But when we recollect that the woman was large, well formed, had a capacious pelvis, and was in her tenth labor, with two small children, we will be better prepared to understand this anomalous presentation.

The death of the second child was caused no doubt, by the separation of its placenta, something like an hour before the child was born. There was no other cause that I can assign for it, for no force sufficient to injure the child was used, and its mal-position was corrected before the contraction of the uterus could have produced its death. Both placentæ were detached from the uterus at the same time.

I am reminded by these occurrences of the first case of twins that ever I encountered, and I will trespass so far on the patience of the Society as to give a brief recital of it.

The subject was a lady, the wife of a neighbor, and the case occurred shortly after I commenced the practice of medicine. The patient was delivered easily, and in good time, of a fine child, and as I was pressed with some other cases, I left her, in the care of an old midwife, doing as well as usual.

I had barely arranged my business at my office, and was on the point of setting off to visit some other patients, when I saw the husband of the lady riding by in a brisk pace. "Halloo!" I called out, "which way?" He laughed, and replied that he "was going over to town to get some things to make *more baby clothes*," his wife having had another child since I left her! I was not a little surprised, nor less mortified at my oversight. However, the family were amiable, and besides were my fast friends, and I never heard of the matter afterwards to my prejudice; but it taught me a useful lesson, which I have never forgotten, which is this—always carefully to examine the abdomen of the parturient woman before the birth of the child, and never leave her until I am satisfied that there is not another.

May, 1845.

